# G-computation of average treatment effects on the treated and the untreated

**DOI:** 10.1186/s12874-016-0282-4

**Published:** 2017-01-09

**Authors:** Aolin Wang, Roch A. Nianogo, Onyebuchi A. Arah

**Affiliations:** 1 Department of Epidemiology, Fielding School of Public Health, University of California, Los Angeles (UCLA), Los Angeles, CA USA; 2 California Center for Population Research (CCPR), Los Angeles, CA USA; 3 UCLA Center for Health Policy Research, Los Angeles, CA USA

**Keywords:** Average treatment effects on the treated (ATT), Average treatment effects on the untreated (ATU), G-computation, Parametric g-formula, Resampling, Simulation

## Abstract

**Background:**

Average treatment effects on the treated (ATT) and the untreated (ATU) are useful when there is interest in: the evaluation of the effects of treatments or interventions on those who received them, the presence of treatment heterogeneity, or the projection of potential outcomes in a target (sub-) population. In this paper we illustrate the steps for estimating ATT and ATU using g-computation implemented via Monte Carlo simulation.

**Methods:**

To obtain marginal effect estimates for ATT and ATU we used a three-step approach: fitting a model for the outcome, generating potential outcome variables for ATT and ATU separately, and regressing each potential outcome variable on treatment intervention.

**Results:**

The estimates for ATT, ATU and average treatment effect (ATE) were of similar magnitude, with ATE being in between ATT and ATU as expected. In our illustrative example, the effect (risk difference [RD]) of a higher education on angina among the participants who indeed have at least a high school education (ATT) was −0.019 (95% CI: −0.040, −0.007) and that among those who have less than a high school education in India (ATU) was −0.012 (95% CI: −0.036, 0.010).

**Conclusions:**

The g-computation algorithm is a powerful way of estimating standardized estimates like the ATT and ATU. Its use should be encouraged in modern epidemiologic teaching and practice.

**Electronic supplementary material:**

The online version of this article (doi:10.1186/s12874-016-0282-4) contains supplementary material, which is available to authorized users.

## Background

In epidemiology, (bio)statistics and related fields, researchers are often interested in the average treatment effect in the total population (average treatment effect, ATE). This quantity provides the average difference in outcome between units assigned to the treatment and units assigned to the placebo (control) [1]. However, in economics and evaluation studies, it has been noted that the average treatment effect among units who actually receive the treatment or intervention (average treatment effects on the treated, ATT) may be the implicit quantity sought and the most relevant to policy makers [2]. For instance, consider a scenario where a government has implemented a smoking cessation campaign intervention to decrease the smoking prevalence in a city and now wishes to evaluate the impact of such intervention. Although the overarching goal of such evaluation may be to assess the impact of such intervention in reducing the prevalence of smoking in the general population (i.e. ATE), researchers and policymakers might be interested in explicitly evaluating the effect of the intervention on those who actually received the intervention (i.e. ATT) but not that on those among whom the intervention was never intended.

Alternatively, researchers may be interested in estimating the potential impact of an existing program in a new target (sub-) population. For instance, one might wish to project the effect of the smoking cessation intervention in a city that did not receive the intervention in order to gauge its potential impact when such intervention is actually implemented. This latter quantity is referred to as the average treatment effect on the untreated (ATU). Interestingly, the ATE can be seen as a weighted average of the ATT and the ATU. All three quantities will be equal when the covariate distribution is the same among the treated and the untreated (e.g. under perfect randomization with perfect compliance or when there is no unmeasured confounders) and there is no effect measure modification by the covariates.

Robins introduced the “g-methods” to estimate such quantities using observational data [3]. Among these, the marginal structural models (MSMs) were designed to estimate marginal quantities (i.e., not conditional on other covariates). The parameters of a MSM can be consistently estimated using two classes of estimators: the g-computation algorithm [4] and the inverse-probability of treatment weighting (IPTW) [5]. G-computation is often seen as a viable alternative to IPTW because g-computation produces more efficient (i.e. small standard errors) and more stable estimates in parametric settings and can better handle heterogeneity involving time-varying exposure and confounding [6]. To date, there are several didactic demonstrations for g-computation [7, 8] and applied examples for projecting the impact of hypothetical interventions aimed at reducing risk factors for coronary heart diseases [9] or diabetes in adult populations [10], or at reducing early childhood adiposity [11]. However, these studies focused on ATE and there are still no accessible demonstrations of g-computation [4] applied to ATT and ATU. This manuscript aims to present an easy-to-use g-computation technique using Monte Carlo simulation for consistently estimating ATT and ATU. We also present alternative ways to obtain ATT and ATU via ATE with sample restriction or g-computation technique without simulation.

## Methods

### Notation and g-computation steps

In the remaining, we will use capital letters to refer to random variables and lowercase letters to represent the specific realizations of the corresponding random variables. Let *A* denote the treatment, with *a* and *a* ^*^ as its index and reference values, *Y* the outcome, ***C*** a set of covariates sufficient for confounding control, and *Y*_*a*_ the potential outcome that would have occurred had treatment *A*, perhaps contrary to fact, been set to *a*. Each subject in the population has a pair of potential outcomes, one being observed and the other being counterfactual. *Y*_*a*_ is the observed outcome had the subject received the treatment *A* = *a* whereas *Y*_a*_ is the counterfactual outcome. Conversely, for subjects who receive placebo (control), *Y*_a*_ is the observed outcome while *Y*_*a*_ is the counterfactual outcome. The ATE, defined as $E\left({Y}_a-{Y}_{a^{*}}\right)$, is the average marginal treatment effect in the total population. The ATT, defined as $E\left({Y}_a-{Y}_{a^{*}}\Big|A=a\right)$ and the ATU, defined as $E\left({Y}_a-{Y}_{a^{*}}\Big|A={a}^{*}\right)$, measure the marginal treatment effect in the subpopulation that received the treatment and the subpopulation that did not, respectively. When the assumptions of consistency [12], conditional exchangeability given ***C*** [13], and positivity [14] are met, the target causal parameters ATE, ATT and ATU on the risk difference scale can be estimated using observational data and the following estimators:

$$ \begin{array}{c}\hfill ATE={\varSigma}_c\left[E\left(Y\Big|A=1,\boldsymbol{C}=\boldsymbol{c}\right)-E\left(Y\Big|A=0,\boldsymbol{C}=\boldsymbol{c}\right)\right]P\left(\boldsymbol{C}=\boldsymbol{c}\right),\hfill \\ {}\hfill ATT=E\left(Y\Big|A=a\right)-{\varSigma}_cE\left(Y\Big|A={a}^{*},\boldsymbol{C}=\boldsymbol{c}\right)P\left(\boldsymbol{C}=\boldsymbol{c}\Big|A=a\right),\kern0.24em \mathrm{and}\hfill \\ {}\hfill ATU={\varSigma}_cE\left(Y\Big|A=a,\boldsymbol{C}=\boldsymbol{c}\right)P\left(\boldsymbol{C}=\boldsymbol{c}\Big|A={a}^{*}\right)-E\left(Y\Big|A={a}^{*}\right).\hfill \end{array} $$

Steps to implement g-computation using Monte Carlo simulation are as follows:

Step 1: Fit a flexible model for *Y* on *A* and covariates ***C*** (i.e. with all possible and relevant interaction terms) and save the regression coefficients.

Step 2: Re-sample the original data with replacement *K* times (e.g. 200 or as many as computationally feasible). Create two copies of this pooled dataset and stack them. Assign a new treatment intervention variable *A = a* for every observation in the first copy and *A = a* ^*^ in the second copy. Then, generate potential outcomes for ATT and ATU separately using the regression coefficients obtained from step 1. For ATT, assign the potential outcome *Y*_*a*_ for *treated* (i.e. *A = a*) individuals as their observed outcome *Y* in the “intervention *A = a*” dataset copy (by consistency), but impute their counterfactual outcome *Y*_a*_ in the “intervention *A = a* ^*^” copy (by conditional exchangeability). This latter counterfactual outcome is simulated under non-treatment, based on the outcome model and regression coefficients from step 1. For ATU, by consistency assumption, assign the potential outcome *Y*_a*_ = *Y* among the *untreated* (i.e. *A = a* ^*^) individuals in the “intervention *A = a* ^*^” dataset copy, and impute their counterfactual outcome *Y*_*a*_ in the “intervention *A = a*” copy. This counterfactual outcome *Y*_*a*_ is simulated under treatment, based on the outcome model and regression coefficients from step 1. Note that the g-computation of the ATT or ATU involves imputing or simulating only half of the potential outcomes under the counterfactual treatment since by consistency under factual treatment the potential outcome is observed.

Step 3: For ATT and ATU respectively, regress the corresponding potential outcome variable on the intervention variable *A* for the entire pooled simulated sample to obtain the point estimate. Repeat steps 1 to 3 on *J* (e.g. 500) bootstrapped samples taken at random with replacement from the original data. We obtain the standard errors (SEs) and 95% confidence intervals (CIs) based on the *J* resultant point estimates from the final regression in step 3. The standard deviation of these *J* point estimates is taken as the standard error and the corresponding 2.5th and 97.5th percentiles are taken as the confidence limits of the 95% CI. Nonparametric bootstrapping [15] can also be used to obtain bias-corrected and accelerated CIs.

One could also obtain ATT and ATU from average treatment effect (ATE) by simply restricting the analysis for ATE estimation to the treated (for ATT) or to the untreated (for ATU) (Additional file 1: Section 1). An alternative g-computation technique without simulation is included in the Additional file 1: Section2.

### Illustrations

We applied the above simulation method to the India sample data from the cross-sectional World Health Survey (WHS) conducted by the WHO from 2002 to 2004 [16]. Samples were probabilistically selected with every individual being assigned to a known non-zero selection probability. All participants were interviewed face-to-face with the standardized WHS survey, which included questions regarding demographic, socioeconomic and behavioral factors. Details of dataset description and variable creation can be found elsewhere [17].

Table 1 displays the estimates for ATT, ATU and ATE on the risk difference and odds ratio scale respectively for binary education (treatment) and binary angina indicator (outcome), accounting for age and gender (covariates). We were interested in estimating the impact of a hypothetical intervention (aimed at ensuring that the target study participants have at least a high school education) on angina diagnosis. The intervention could be implemented (i) universally in the whole population of India (ATE), (ii) among individuals of a sub-population of India who actually completed high school or had higher educational attainment (ATT), or (iii) among individuals of a sub-population of India who had less than a high school education (ATU) when the survey was conducted. Detailed steps and the accompanying SAS codes for this illustrative example are included in the Additional file 1: Section 3 and Additional file 1: Section 5.

**Table 1:** Effect estimates obtained from g-computation using the illustrative example dataset^a^ (*N* = 7706)

	G-computation (via Monte Carlo Simulation)^**b**^		
	Point Estimate	Standard Error	95% Confidence Interval
Average Treatment Effect among the Treated (ATT)			
Risk difference	−0.019	0.009	−0.040, −0.007
Odds ratio	0.773	0.112	0.607, 0.944
Average Treatment Effect among the Untreated (ATU)			
Risk difference	−0.012	0.012	−0.036, 0.010
Odds ratio	0.910	0.133	0.678, 1.177
Average Treatment Effect (ATE)			
Risk difference	−0.015	0.011	−0.036, 0.007
Odds ratio	0.884	0.127	0.676, 1.130

^a^Treatment: education (1 = high school and beyond, 0 = less than high school); outcome: ever diagnosed with angina (1 = yes, 0 = no); covariates: age and gender

^b^The outcome model included all possible 2- and 3-way product terms between education and covariates. Standard errors and the 95% confidence limits were based on 500 bootstrap samples where the standard deviation of the 500 point estimates was taken as the standard error and the corresponding 2.5th and 97.5th percentiles were taken as the lower and upper limit of the 95% confidence interval

## Results

In the illustration, participants with at least a high school education were less likely to report having an angina diagnosis compared to those with less than a high school education, based on both risk difference (RD) and odds ratio (OR) measures (Table 1). The estimates for ATT, ATU and ATE were of similar magnitude, with ATE being in between ATT and ATU as expected. The ATT estimates were of slightly greater magnitude (RD: −0.019, 95% CI: −0.040, −0.007; OR: 0.773, 95% CI: 0.607, 0.944) than the ATU estimates (RD: −0.012, 95% CI: −0.036, 0.010; OR: 0.910, 95% CI: 0.678, 1.177), suggesting that the protective effect of a higher education on angina may be stronger among the participants who indeed have at least a high school education than among those who have less than a high school education in India.

Similar results obtained via g-computation without simulation are presented in the Additional file 1: Table S1.

## Discussions

In this article, we presented a 3-step approach to estimating ATT and ATU via Monte Carlo simulation. Since ATE risk difference is the weighted average of ATT and ATU, weighted by the relative sample size of those who are treated and untreated, ATT and ATU can also be estimated from ATE via sample restriction.

When generating the potential outcomes in step 2, the potential outcome will be the same as the observed outcome if the intervention assignment (e.g. treatment) is indeed what the subject originally received and the consistency assumption is satisfied. Accordingly, the counterfactual outcome for the same subjects will be imputed (simulated) based on the outcome from those who received the alternative to treatment (e.g. placebo) and are comparable (i.e., exchangeable) conditional on measured covariates or confounders, if the assumption of conditional exchangeability assumption is met. In step 2 of the alternative g-computation approach that does not require simulation, the predicted outcomes [i.e., *E*(*Y*|*A* = *a*, ***C*** = ***c***)] are generated for both treated and untreated individuals. While the approach via simulation clearly demonstrates the importance of the two core assumptions—consistency and conditional exchangeability—to estimate causal parameters from observational data, the approach without simulation is less computationally intensive.

We also need the positivity assumption which requires that there exist participants who experienced all levels of the treatment (such as being treated or untreated) for every combination of the values of the observed confounders in the population under study [14]. This latter assumption needs to be supported by the data at hand. Steps for implementing g-computation for ATT and ATU allow us to better understand the importance of assumptions that are often listed but seldom discussed.

Besides the consistency, conditional exchangeability and positivity assumptions, other implicit assumptions such as the absence of other biases (selection bias and measurement error) and correct model specification need to be satisfied in order to estimate ATE, ATT and ATU consistently. G-computation relies heavily on outcome model specification as shown in the above steps, in which we used the regression coefficients we obtained from the outcome regression model in step 1 to predict potential outcomes. On the contrary, the IPTW method relies on correct exposure model specification assumptions. Therefore, these two g-methods can sometimes yield different results. Their strengths and limitations, and performance under violation of the positivity assumption have also been discussed in the literature [6, 18]. When possible, researchers could use both methods, or use doubly robust methods [19–21] where consistent estimates for the target effects can be obtained as long as either the outcome or exposure model is correctly specified.

## Conclusion

The g-computation algorithm is a powerful way of estimating standardized estimates like the ATT and ATU, beyond routine age- and sex-standardization and as an alternative to IPTW fitting of MSM [22]. It should be used in modern epidemiologic teaching and practice.

## Additional file

Additional file 1: In the supplementary file, we presented two alternative ways for estimating ATT and ATU, and the detailed g-computation steps, and the corresponding SAS code for the illustration. (DOCX 81 kb)

## Abbreviations

ATE: Average treatment effect
ATT: Average treatment effects on the treated
ATU: Average treatment effects on the untreated
MSMs: Marginal structural models
