# Supplementary material for: G-computation of average treatment effects on the treated and the untreated
Source: BMC Med Res Methodol. 2017 Jan 9;17:3. doi: 10.1186/s12874-016-0282-4 (PMC5223318; doi:10.1186/s12874-016-0282-4)
Supplement: Additional file 1: — In the supplementary file, we presented two alternative ways for estimating ATT and ATU, and the detailed g-computation steps, and the corresponding SAS code for the illustration. (DOCX 81 kb) [file 12874_2016_282_MOESM1_ESM.docx]

**­G-Computation of Average Treatment Effects on the Treated and the Untreated**

**Online supplementary materials**

Aolin Wang^1,2^*, Roch A. Nianogo^1,2^, Onyebuchi A. Arah^1-3^

^1^Department of Epidemiology, Fielding School of Public Health, University of California, Los Angeles (UCLA), Los Angeles, California, USA

^2^California Center for Population Research (CCPR), Los Angeles, California, USA

^3^UCLA Center for Health Policy Research, Los Angeles, California, USA

*Corresponding author. Department of Epidemiology, UCLA Fielding School of Public Health, 650 Charles E. Young Drive South, Los Angeles, CA 90095-1772, USA. Email: aolinw@ucla.edu

**Abbreviations**

ATE: average treatment effect

ATT: average treatment effects on the treated

ATU: average treatment effects on the untreated

MSMs: marginal structural models

Contents

[1. Simplified way to obtain ATT and ATU based on ATE with restriction 1](#_Toc464232197)

[2. G-computation without simulation 2](#_Toc464232198)

[3. G-computation steps (via simulation) for the illustration 3](#_Toc464232199)

[4. Supplementary table 5](#_Toc464232200)

[5. SAS code for illustrative example using g-computation of marginal structural models 6](#_Toc464232201)

[1. G-computation via Monte Carlo simulation 7](#_Toc464232202)

[Risk difference 7](#_Toc464232203)

[Odds ratio 13](#_Toc464232204)

[2. G-computation without simulation 21](#_Toc464232205)

[Risk difference 21](#_Toc464232206)

[Odds ratio 23](#_Toc464232207)

# **1. Simplified way to obtain ATT and ATU based on ATE with restriction**

For a binary treatment variable *A*, we use 1 and 0 to denote treatment and no treatment respectively. ATE is the weighted average of ATT and ATU, weighted by the relative sample size of those who are treated and untreated. When the assumptions of consistency [5], conditional exchangeability given C [6], and positivity [7] are met, ATE (on the risk difference scale) can be expressed in terms of observable quantities (i.e. using observational data):

$ATE =\sum_{c} [E\left( Y | A=1,\boldsymbol{C=c} \right)-E\left( Y | A=0,\boldsymbol{C=c} \right)]P\left( \boldsymbol{C=c} \right)$

$=\sum_{c,a} [E\left( Y | A=1,\boldsymbol{C=c} \right)-E\left( Y | A=0,\boldsymbol{C=c} \right)]P\left( \boldsymbol{C=c|}A=a \right)P(A=a)$

$=\left\{ \sum_{c} [E\left( Y | A=1,\boldsymbol{C=c} \right)-E\left( Y | A=0,\boldsymbol{C=c} \right)]P\left( \boldsymbol{C=c} | A=1 \right) \right\}P\left( A=1 \right)$

$\boldsymbol{+ \{}\sum_{c} [E\left( Y | A=1,\boldsymbol{C=c} \right)-E\left( Y | A=0,\boldsymbol{C=c} \right)]P\left( \boldsymbol{C=c} | A=0 \right)\}P\left( A=0 \right)$.

Hence,

$ATE=ATT\cdot P\left( A=1 \right)\boldsymbol{+}ATU\cdot P\left( A=0 \right)$.

Without sample restriction, $P\left( A=1 \right)$ and $P\left( A=0 \right)$ will be the observed prevalence of treatment or no treatment and hence we obtain ATE. By restricting the analytical sample to the treated ($A=1$), we assign $P\left( A=1 \right)\boldsymbol{=}1$ and $P\left( A=0 \right)\boldsymbol{=}0$ and hence obtain ATT. On the contrary, by restricting the analytical sample to the untreated ($A=0$), we assign $P\left( A=1 \right)\boldsymbol{=}0$ and $P\left( A=0 \right)\boldsymbol{=}1$ and hence obtain ATU.

# **2. G-computation without simulation**

Steps to implement g-computation without simulation (using predicted conditional probabilities of the outcome) are as follows:

Step 1: Use bootstrap to resample the original data set *J* (e.g. 1000) times. For each bootstrapped sample, fit a flexible model for *Y* conditional on *A* and covariates ***C*** that accounts for possible treatment heterogeneity by covariates (i.e. with all possible and relevant interaction terms) and save the parameters.

Step 2: Compute two predicted potential outcomes under treatment (setting *A* = 1) and no treatment (setting *A* = 0) for every individual, using the parameters from step 1.

Step 3: For each of the *J* bootstrap samples, take the contrast (i.e. risk difference or odds ratio) between the predicted potential outcomes under treatment and the predicted potential outcomes under no treatment among the treated (for ATT) and among the untreated (for ATU) respectively. Summarize such contrast across *J* bootstrap samples and take the mean and standard deviation (SD) as the point estimate and standard error for marginal ATT and ATU. The corresponding Wald type 95% confidence interval (CI) was calculated as: point estimate ± 1.96 × SD. Notice that by summarizing the contrast between the predicted potential outcomes under treatment versus no treatment for the entire sample (without restriction), we can obtain the estimates for marginal ATE.

After applying the g-computation via resampling method to the illustrative dataset, we obtained similar results as the ones presented in the main text (Table S1). The corresponding SAS code is provided at the end of this supplementary document.

# **3. G-computation steps (via simulation) for the illustration**

For the illustrative example, we implemented the following steps to estimate the average effect of education on angina among people with high school and beyond education (ATT) and among people with less than high school education (ATU), using g-computation via simulation:

Step 1: Fit a model for angina indicator conditional on binary education indicator and covariates including age and gender, including all possible 2- and 3-way product terms between education and covariates. Save the parameters.

Step 2: Re-sample the original data with replacement *K* (e.g. 50 or more) times. Create two copies of this pooled dataset and stack them. Assign a new education intervention variable *A =* 1 (education= high school and beyond) to every observation in the first copy and *A =* 0 education = ‘less than high school’ in the second copy. Then, generate potential outcomes for estimating ATT and ATU separately.

For ATT, assign the potential outcome for individuals with high school and beyond education as their observed outcome in the “intervention *A =* 1” copy (by consistency), but impute their counterfactual outcome in the “intervention *A =* 0”copy (by conditional exchangeability). This latter counterfactual outcome is simulated under less than high school education (assigning education to be 0), based on the model and parameters from step 1.

For ATU, assign the potential outcome for individuals with less than high school education as their observed outcome in the “intervention *A =* 0” copy but impute their counterfactual outcome in the “intervention *A =* 1” copy. This latter counterfactual outcome is simulated under high school and beyond education (assigning education to be 1), based on the model and parameters from step 1.

Step 3: For ATT and ATU respectively, regress the corresponding potential outcome variable on the intervention variable *Education* for the entire pooled simulated sample to obtain the point estimate. Repeat steps 1 to 3 on 500 bootstrap samples taken at random with replacement from the original data. We obtain the standard errors and 95% confidence intervals (CIs) based on the 500 resultant point estimates from the final regression in step 3. The standard deviation of these 500 point estimates is taken as the standard error and the corresponding 2.5^th^ and 97.5^th^ percentiles are taken as the confidence limits of the 95% CI. The Wald type confidence intervals (CIs) for marginal ATT and ATU of education on angina can be calculated as: point estimate ± 1.96 × SD, where SD was the standard deviation of the 500 resultant point estimates from the final regression in step 3.

# **4. Additional file 1: Table S1**

| **Table S1.** Effect estimates obtained from g-computation without simulation**^a^** using the illustrative dataset**^b^** (N= 7706) | | | |
| --- | --- | --- | --- |
|  | G-computation without simulation | | |
|  | Point Estimate | Standard Error | 95% Confidence Interval |
| Average Treatment Effect among the Treated (ATT) | | | |
| Risk difference | -0.021 | 0.008 | -0.037, -0.004 |
| Odds ratio | 0.732 | 0.082 | 0.581, 0.891 |
| Average Treatment Effect among the Untreated (ATU) | | | |
| Risk difference | -0.013 | 0.012 | -0.035, 0.011 |
| Odds ratio | 0.886 | 0.126 | 0.669, 1.163 |
| Average Treatment Effect (ATE) | | | |
| Risk difference | -0.015 | 0.011 | -0.035, 0.007 |
| Odds ratio | 0.858 | 0.114 | 0.655, 1.101 |
| ^a^ The outcome model included all possible 2- and 3-way product terms between education and covariates. Effect estimates and bootstrap confidence intervals (percentile) were based on 1000 bootstrap samples.  ^b^ Treatment: education (1=high school and beyond, 0=less than high school); outcome: ever diagnosed with angina (1=yes, 0=no); covariates: age and gender. | | | |

#

# **5. SAS code for illustrative example using g-computation of marginal structural models**

/***************************************************************************************************

Title: G-Computation of Average Treatment Effects on the Treated and the Untreated

Purpose: To illustrate g-computation techniques for consistently estimating ATT and ATU

Author: Aolin Wang <aolinw@ucla.edu> & Roch Nianogo <

niaroch@ucla.edu>

Contents: 1. G-computation via Monte Carlo simulation

(simulate the individual counterfactual outcomes,

method described in the main manuscript)

2. G-computation without simulation

(predict the conditional probability of the outcome,

method described in the supplementary file)

Notation:

Y = Angina (1=yes, 0=no)

A = Education (1=high school and beyond, 0=less than high school)

C1 = Gender (1=female, 0=male)

C2 = Recentered and rescaled Age

dsn = India Dataset

****************************************************************************************************/

Options pageno=**1** nodate symbolgen formdlim = '-'; ods graphics off;

/*DATA*/

libname d "Assign the library path here";

**data** dsn; set d.india; **run**; /*read in dataset*/

/********************************************************************

## 1. G-computation via Monte Carlo simulation

********************************************************************/

### /*****RISK DIFFERENCE*****/

/*

Step 1: Fit a flexible model for Y on A and covariates C and save the parameters

*/

**proc** **genmod** data=dsn desc;

model Y = A | C1 | C2/link=id d=bin;

ods output ParameterEstimates=dsn_parm_RD (keep = parameter estimate);

**run**;

**proc** **transpose** data=dsn_parm_RD out=dsn_parm_RD1 (drop = _NAME_ brd_Scale) prefix=brd_;

id parameter;

var estimate;

**run**;

**Data** dsn_parm_RD2;

set dsn_parm_RD1;

m = **1**;

**data** dsn;

set dsn;

m = **1**;

**run**;

**proc** **sort** data=dsn_parm_RD2; by m;

**proc** **sort** data=dsn; by m;

**data** gdsn_parm_RD3;

merge dsn dsn_parm_RD2;

by m;

**run**;

/*

Step 2: Create two copies of the original dataset and stack them

*/

*sample with replacement from data (to reduce Monte Carlo error, can increase the rep number);

**proc** **surveyselect** data=gdsn_parm_RD3 out=gc_MC_RD

seed = **7080** method = urs

samprate = **1** outhits rep = **50**;

**run**;

**Data** gcdsn_RD;

set gc_MC_RD;

doA = **0**;

output;

set gc_MC_RD;

doA = **1**;

output;

**run**;

**proc** **datasets** lib=work memtype=data;

delete dsn_parm_RD dsn_parm_RD1 dsn_parm_RD2 gdsn_parm_RD3 gc_MC_RD;

**run**; **quit**;

/*

Step 2: Generate potential outcomes for ATT and ATU separately

*/

**Data** gcdsn_RD;

set gcdsn_RD;

call streaminit(**1321**);

/*ATT*/

if A = **1** and doA = **1** then Y_att_rd = Y; /*Observed outcome*/

if A = **1** and doA = **0** then Y_att_rd = rand('bernoulli', brd_Intercept + brd_A***0** + brd_C1*C1 +

brd_C2*C2 + brd_A_C2***0***C2 +

brd_A_C1***0***C1 + brd_C1_C2*C2*C1 +

brd_A_C1_C2***0***C2*C1); /*Counterfactual outcome*/

/*ATU*/

if A = **0** and doA = **1** then Y_atu_rd = rand('bernoulli', brd_Intercept + brd_A***1** + brd_C1*C1 +

brd_C2*C2 + brd_A_C2***1***C2 +

brd_A_C1***1***C1 + brd_C1_C2*C2*C1 +

brd_A_C1_C2***1***C2*C1);/*Counterfactual outcome*/

if A = **0** and doA = **0** then Y_atu_rd = Y;/*Observed outcome*/

/*ATE*/

if doA = **0** then Y_ate_rd = rand('bernoulli', brd_Intercept + brd_A***0** + brd_C1*C1 +

brd_C2*C2 + brd_A_C2***0***C2 +

brd_A_C1***0***C1 + brd_C1_C2*C2*C1 +

brd_A_C1_C2***0***C2*C1); /*Counterfactual outcome*/

if doA = **1** then Y_ate_rd = rand('bernoulli', brd_Intercept + brd_A***1** + brd_C1*C1 +

brd_C2*C2 + brd_A_C2***1***C2 +

brd_A_C1***1***C1 + brd_C1_C2*C2*C1 +

brd_A_C1_C2***1***C2*C1); /*Counterfactual outcome*/

output;

**run**;

/*

Step 3: Regress the corresponding potential outcome variable on the intervention variable A

*/

*3a) point estimate: regress potential outcome on intervention A using the pooled data;

ods listing close;

**proc** **genmod** data=gcdsn_RD desc;

Title "ATT_RD";

model Y_att_rd = doA /link=id d=bin;

ods output ParameterEstimates=parm_ATT_RD(keep = parameter estimate);

**run**;

**proc** **genmod** data=gcdsn_RD desc;

Title "ATU_RD";

model Y_atu_rd = doA /link=id d=bin;

ods output ParameterEstimates=parm_ATU_RD(keep = parameter estimate);

**run**;

**proc** **genmod** data=gcdsn_RD desc;

Title "ATE_RD";

model Y_ate_rd = doA /link=id d=bin;

ods output ParameterEstimates=parm_ATE_RD(keep = parameter estimate);

**run**;

*3b) SE: bootstrap [repeat Step 1 - 3a) 500 times and summarize the resultant estimates];

**proc** **surveyselect** data=dsn out=gc_boot_RD

seed = **107080** method = urs

samprate = **1** outhits rep = **500**;

**run**;

*repeat step 1;

**proc** **genmod** data=gc_boot_RD desc;

by replicate;

model Y = A | C1 | C2/link=id d=bin;

ods output ParameterEstimates=dsn_parm_RD (keep = replicate parameter estimate);

**run**;

**proc** **transpose** data=dsn_parm_RD out=dsn_parm_RD1 (drop = _NAME_ brd_Scale) prefix=brd_;

by replicate;

id parameter;

var estimate;

**run**;

**proc** **sort** data=dsn_parm_RD1; by replicate;

**proc** **sort** data=gc_boot_RD; by replicate; **run**;

**data** boot_RD;

merge gc_boot_RD dsn_parm_RD1;

by replicate;

rename replicate = bootrep;

drop NumberHits;

**run**;

*repeat step 2;

**proc** **surveyselect** data=boot_RD out=boot_RD2

seed = **207080** method = urs

samprate = **1** outhits rep = **50**;

**run**;

**Data** boot_RD3;

set boot_RD2;

doA = **0**;

output;

set boot_RD2;

doA = **1**;

output;

**run**;

**proc** **datasets** lib=work memtype=data;

delete dsn_parm_RD dsn_parm_RD1 dsn gcdsn_RD gc_boot_RD boot_RD boot_RD2;

**run**; **quit**;

**Data** boot_RD3;

set boot_RD3;

call streaminit(**11321**);

/*ATT*/

if A = **1** and doA = **1** then Y_att_rd = Y; /*Observed outcome*/

if A = **1** and doA = **0** then Y_att_rd = rand('bernoulli', brd_Intercept + brd_A***0** + brd_C1*C1 +

brd_C2*C2 + brd_A_C2***0***C2 +

brd_A_C1***0***C1 + brd_C1_C2*C2*C1 +

brd_A_C1_C2***0***C2*C1); /*Counterfactual outcome*/

/*ATU*/

if A = **0** and doA = **1** then Y_atu_rd = rand('bernoulli', brd_Intercept + brd_A***1** + brd_C1*C1 +

brd_C2*C2 + brd_A_C2***1***C2 +

brd_A_C1***1***C1 + brd_C1_C2*C2*C1 +

brd_A_C1_C2***1***C2*C1);/*Counterfactual outcome*/

if A = **0** and doA = **0** then Y_atu_rd = Y;/*Observed outcome*/

/*ATE*/

if doA = **0** then Y_ate_rd = rand('bernoulli', brd_Intercept + brd_A***0** + brd_C1*C1 +

brd_C2*C2 + brd_A_C2***0***C2 +

brd_A_C1***0***C1 + brd_C1_C2*C2*C1 +

brd_A_C1_C2***0***C2*C1); /*Counterfactual outcome*/

if doA = **1** then Y_ate_rd = rand('bernoulli', brd_Intercept + brd_A***1** + brd_C1*C1 +

brd_C2*C2 + brd_A_C2***1***C2 +

brd_A_C1***1***C1 + brd_C1_C2*C2*C1 +

brd_A_C1_C2***1***C2*C1); /*Counterfactual outcome*/

output;

**run**;

**proc** **sort** data=boot_RD3; by bootrep; **run**;

/*ATT_RD*/

**proc** **genmod** data=boot_RD3 desc;

by bootrep;

model Y_att_rd = doA/link=id d=bin;

ods output ParameterEstimates=gc_boot_ATT_RD (keep = bootrep Parameter Estimate);

**run**;

/*ATU_RD*/

**proc** **genmod** data=boot_RD3 desc;

by bootrep;

model Y_atu_rd = doA/link=id d=bin;

ods output ParameterEstimates=gc_boot_ATU_RD (keep = bootrep Parameter Estimate);

**run**;

/*ATE_RD*/

**proc** **genmod** data=boot_RD3 desc;

by bootrep;

model Y_ate_rd = doA/link=id d=bin;

ods output ParameterEstimates=gc_boot_ATE_RD (keep = bootrep Parameter Estimate);

**run**;

**proc** **datasets** lib=work memtype=data;

delete boot_RD3;

**run**; **quit**;

**proc** **univariate** data=gc_boot_ATT_RD noprint;

where parameter = "doA";

var Estimate;

output out=summ_ATT_RD

pctlpts=**2.5** **50** **97.5**

pctlpre =ATT_RD_p

mean =Mean_ATT_RD

std =SD_ATT_RD;

**run**;

**proc** **univariate** data=gc_boot_ATU_RD noprint;

where parameter = "doA";

var Estimate;

output out=summ_ATU_RD

pctlpts=**2.5** **50** **97.5**

pctlpre =ATU_RD_p

mean =Mean_ATU_RD

std =SD_ATU_RD;

**run**;

**proc** **univariate** data=gc_boot_ATE_RD noprint;

where parameter = "doA";

var Estimate;

output out=summ_ATE_RD

pctlpts=**2.5** **50** **97.5**

pctlpre =ATE_RD_p

mean =Mean_ATE_RD

std =SD_ATE_RD;

**run**;

*combine point estimates for ATT, ATU, and ATE;

**proc** **transpose** data=Parm_att_rd out=ATT_RD_ptest (keep=ATT_RD_doA) prefix=ATT_RD_;

id parameter;

var estimate;

**run**;

**proc** **transpose** data=Parm_atu_rd out=ATU_RD_ptest (keep=ATU_RD_doA) prefix=ATU_RD_;

id parameter;

var estimate;

**run**;

**proc** **transpose** data=Parm_ate_rd out=ATE_RD_ptest (keep=ATE_RD_doA) prefix=ATE_RD_;

id parameter;

var estimate;

**run**;

**data** result;

merge ATT_RD_ptest ATU_RD_ptest ATE_RD_ptest

summ_ATT_RD summ_ATU_RD summ_ATE_RD;

rename ATT_RD_doA = ATT_RD_ptest

ATU_RD_doA = ATU_RD_ptest

ATE_RD_doA = ATE_RD_ptest;

**run**;

ods listing;

**proc** **print** data = result;

var ATT_RD_ptest SD_ATT_RD

ATU_RD_ptest SD_ATU_RD

ATE_RD_ptest p50 SD_ATE_RD;

**run**;

### /*****ODDS RATIO*****/

/*

Step 1: Fit a flexible model for Y on A and covariates C and save the parameters

*/

**proc** **genmod** data=dsn desc;

model Y = A | C1 | C2/link=logit d=bin;

ods output ParameterEstimates=dsn_parm_OR (keep = parameter estimate);

**run**;

**proc** **transpose** data=dsn_parm_OR out=dsn_parm_OR1 (drop = _NAME_ bOR_Scale) prefix=bOR_;

id parameter;

var estimate;

**run**;

**Data** dsn_parm_OR2;

set dsn_parm_OR1;

m = **1**;

**run**;

**data** dsn;

set dsn;

m = **1**;

**run**;

**proc** **sort** data=dsn_parm_OR2; by m;

**proc** **sort** data=dsn; by m;

**data** gdsn_parm_OR3;

merge dsn dsn_parm_OR2;

by m;

**run**;

/*

Step 2: Create two copies of the original dataset and stack them

*/

*sample with replacement from data (to reduce Monte Carlo error);

**proc** **surveyselect** data=gdsn_parm_OR3 out=gc_MC_OR

seed = **5060** method = urs

samprate = **1** outhits rep = **50**;

**run**;

**Data** gcdsn_OR;

set gc_MC_OR;

doA = **0**;

output;

set gc_MC_OR;

doA = **1**;

output;

**run**;

**proc** **datasets** lib=work memtype=data;

delete dsn_parm_OR dsn_parm_OR1 dsn_parm_OR2 gdsn_parm_OR3 gc_MC_OR;

**run**; **quit**;

/*

Step 2: Generate potential outcomes for ATT and ATU separately

*/

**Data** gcdsn_OR;

set gcdsn_OR;

call streaminit(**3321**);

/*ATT*/

if A = **1** and doA = **1** then Y_att_OR = Y; /*Observed outcome*/

if A = **1** and doA = **0** then Y_att_OR = rand('bernoulli', (**1**/(**1** + exp(-(bOR_Intercept + bOR_A***0** + bOR_C1*C1

+bOR_C2*C2 + bOR_A_C2***0***C2 +

bOR_A_C1***0***C1 + bOR_C1_C2*C2*C1 +

bOR_A_C1_C2***0***C2*C1))))); /*Counterfactual outcome*/

/*ATU*/

if A = **0** and doA = **1** then Y_atu_OR = rand('bernoulli', (**1**/(**1** + exp(-(bOR_Intercept + bOR_A***1** + bOR_C1*C1

+bOR_C2*C2 + bOR_A_C2***1***C2 +

bOR_A_C1***1***C1 + bOR_C1_C2*C2*C1 +

bOR_A_C1_C2***1***C2*C1)))));/*Counterfactual outcome*/

if A = **0** and doA = **0** then Y_atu_OR = Y;/*Observed outcome*/

/*ATE*/

if doA = **0** then Y_ate_OR = rand('bernoulli', (**1**/(**1** + exp(-(bOR_Intercept + bOR_A***0** + bOR_C1*C1

+bOR_C2*C2 + bOR_A_C2***0***C2 +

bOR_A_C1***0***C1 + bOR_C1_C2*C2*C1 +

bOR_A_C1_C2***0***C2*C1))))); /*Counterfactual outcome*/

if doA = **1** then Y_ate_OR = rand('bernoulli', (**1**/(**1** + exp(-(bOR_Intercept + bOR_A***1** + bOR_C1*C1

+bOR_C2*C2 + bOR_A_C2***1***C2 +

bOR_A_C1***1***C1 + bOR_C1_C2*C2*C1 +

bOR_A_C1_C2***1***C2*C1))))); /*Counterfactual outcome*/

output;

**run**;

/*

Step 3: Regress the corresponding potential outcome variable on the intervention variable A

*/

*3a) point estimate: regress potential outcome on intervention A using the pooled data;

ods listing close;

**proc** **genmod** data=gcdsn_OR desc;

Title "ATT_OR";

model Y_att_OR = doA /link=logit d=bin;

ods output ParameterEstimates=parm_ATT_OR(keep = parameter estimate);

**run**;

**proc** **genmod** data=gcdsn_OR desc;

Title "ATU_OR";

model Y_ATU_OR = doA /link=logit d=bin;

ods output ParameterEstimates=parm_ATU_OR(keep = parameter estimate);

**run**;

**proc** **genmod** data=gcdsn_OR desc;

Title "ATE_OR";

model Y_ATE_OR = doA /link=logit d=bin;

ods output ParameterEstimates=parm_ATE_OR(keep = parameter estimate);

**run**;

*3b) SE: bootstrap [repeat Step 1 - 3a) 500 times and summarize the resultant estimates];

**proc** **surveyselect** data=dsn out=gc_boot_OR

seed = **15340** method = urs

samprate = **1** outhits rep = **500**;

**run**;

*repeat step 1;

**proc** **genmod** data=gc_boot_OR desc;

by replicate;

model Y = A | C1 | C2/link=logit d=bin;

ods output ParameterEstimates=dsn_parm_OR (keep = replicate parameter estimate);

**run**;

**proc** **transpose** data=dsn_parm_OR out=dsn_parm_OR1 (drop = _NAME_ bOR_Scale) prefix=bOR_;

by replicate;

id parameter;

var estimate;

**run**;

**proc** **sort** data=dsn_parm_OR1; by replicate;

**proc** **sort** data=gc_boot_OR; by replicate; **run**;

**data** boot_OR;

merge gc_boot_OR dsn_parm_OR1;

by replicate;

rename replicate = bootrep;

drop NumberHits;

**run**;

*repeat step 2;

**proc** **surveyselect** data=boot_OR out=boot_OR2

seed = **25340** method = urs

samprate = **1** outhits rep = **50**;

**run**;

**Data** boot_OR3;

set boot_OR2;

doA = **0**;

output;

set boot_OR2;

doA = **1**;

output;

**run**;

**proc** **datasets** lib=work memtype=data;

delete dsn_parm_OR dsn_parm_OR1 dsn gcdsn_OR gc_boot_OR boot_OR boot_OR2;

**run**; **quit**;

**Data** boot_OR3;

set boot_OR3;

call streaminit(**13321**);

/*ATT*/

if A = **1** and doA = **1** then Y_att_OR = Y; /*Observed outcome*/

if A = **1** and doA = **0** then Y_att_OR = rand('bernoulli', (**1**/(**1** + exp(-(bOR_Intercept + bOR_A***0** + bOR_C1*C1

+bOR_C2*C2 + bOR_A_C2***0***C2 +

bOR_A_C1***0***C1 + bOR_C1_C2*C2*C1 +

bOR_A_C1_C2***0***C2*C1))))); /*Counterfactual outcome*/

/*ATU*/

if A = **0** and doA = **1** then Y_atu_OR = rand('bernoulli', (**1**/(**1** + exp(-(bOR_Intercept + bOR_A***1** + bOR_C1*C1

+bOR_C2*C2 + bOR_A_C2***1***C2 +

bOR_A_C1***1***C1 + bOR_C1_C2*C2*C1 +

bOR_A_C1_C2***1***C2*C1)))));/*Counterfactual outcome*/

if A = **0** and doA = **0** then Y_atu_OR = Y;/*Observed outcome*/

/*ATE*/

if doA = **0** then Y_ate_OR = rand('bernoulli', (**1**/(**1** + exp(-(bOR_Intercept + bOR_A***0** + bOR_C1*C1

+bOR_C2*C2 + bOR_A_C2***0***C2 +

bOR_A_C1***0***C1 + bOR_C1_C2*C2*C1 +

bOR_A_C1_C2***0***C2*C1))))); /*Counterfactual outcome*/

if doA = **1** then Y_ate_OR = rand('bernoulli', (**1**/(**1** + exp(-(bOR_Intercept + bOR_A***1** + bOR_C1*C1

+bOR_C2*C2 + bOR_A_C2***1***C2 +

bOR_A_C1***1***C1 + bOR_C1_C2*C2*C1 +

bOR_A_C1_C2***1***C2*C1))))); /*Counterfactual outcome*/

output;

**run**;

**proc** **sort** data=boot_OR3; by bootrep; **run**;

/*ATT_OR*/

**proc** **genmod** data=boot_OR3 desc;

by bootrep;

model Y_att_OR = doA/link=logit d=bin;

ods output ParameterEstimates=gc_boot_ATT_OR (keep = bootrep Parameter Estimate);

**run**;

/*ATU_OR*/

**proc** **genmod** data=boot_OR3 desc;

by bootrep;

model Y_atu_OR = doA/link=logit d=bin;

ods output ParameterEstimates=gc_boot_ATU_OR (keep = bootrep Parameter Estimate);

**run**;

/*ATE_OR*/

**proc** **genmod** data=boot_OR3 desc;

by bootrep;

model Y_ate_OR = doA/link=logit d=bin;

ods output ParameterEstimates=gc_boot_ATE_OR (keep = bootrep Parameter Estimate);

**run**;

**proc** **datasets** lib=work memtype=data;

delete boot_OR3;

**run**; **quit**;

**proc** **univariate** data=gc_boot_ATT_OR noprint;

where parameter = "doA";

var Estimate;

output out=summ_ATT_OR

pctlpts=**2.5** **50** **97.5**

pctlpre =ATT_OR_p

mean =Mean_ATT_OR

std =SD_ATT_OR;

**run**;

**proc** **univariate** data=gc_boot_ATU_OR noprint;

where parameter = "doA";

var Estimate;

output out=summ_ATU_OR

pctlpts=**2.5** **50** **97.5**

pctlpre =ATU_OR_p

mean =Mean_ATU_OR

std =SD_ATU_OR;

**run**;

**proc** **univariate** data=gc_boot_ATE_OR noprint;

where parameter = "doA";

var Estimate;

output out=summ_ATE_OR

pctlpts=**2.5** **50** **97.5**

pctlpre =ATE_OR_p

mean =Mean_ATE_OR

std =SD_ATE_OR;

**run**;

*combine point estimates for ATT, ATU, and ATE;

**proc** **transpose** data=Parm_att_OR out=ATT_OR_ptest (keep=ATT_OR_doA) prefix=ATT_OR_;

id parameter;

var estimate;

**run**;

**proc** **transpose** data=Parm_atu_OR out=ATU_OR_ptest (keep=ATU_OR_doA) prefix=ATU_OR_;

id parameter;

var estimate;

**run**;

**proc** **transpose** data=Parm_ate_OR out=ATE_OR_ptest (keep=ATE_OR_doA) prefix=ATE_OR_;

id parameter;

var estimate;

**run**;

**data** result;

merge ATT_OR_ptest ATU_OR_ptest ATE_OR_ptest

summ_ATT_OR summ_ATU_OR summ_ATE_OR;

rename ATT_OR_doA = ATT_OR_ptest

ATU_OR_doA = ATU_OR_ptest

ATE_OR_doA = ATE_OR_ptest;

**run**;

*exponentiate the log(OR);

**data** result; set result;

array e[**15**] ATT_OR_ptest ATU_OR_ptest ATE_OR_ptest Mean_ATT_OR Mean_ATU_OR Mean_ATE_OR

ATT_OR_p2_5 ATU_OR_p2_5 ATE_OR_p2_5 ATT_OR_p50 ATU_OR_p50 ATE_OR_p50

ATT_OR_p97_5 ATU_OR_p97_5 ATE_OR_p97_5;

do i=**1** to **15**; e[i] = exp(e[i]); end;

drop i;

**run**;

ods listing;

**proc** **print** data = result;

var ATT_OR_ptest SD_ATT_OR

ATU_OR_ptest SD_ATU_OR

ATE_OR_ptest SD_ATE_OR;

**run**;

/******************* End of G-computation simulation ******************/

/********************************************************************

## 2. G-computation without simulation

********************************************************************/

### /*****RISK DIFFERENCE*****/

/*

Step 1: Use bootstrap to resample the original data set 1000 times

*/

**proc** **surveyselect** data=dsn out=dsn_boot_RD

seed = **1321** method = urs

samprate = **1** outhits rep = **1000**;

**run**;

**proc** **sort** data=dsn_boot_RD; by replicate id; **run**;

/*

Step 1: Fit a flexible model for Y on A and covariates C for each bootstrapped sample

*/

ods listing close;

**proc** **genmod** data=dsn_boot_RD desc;

by replicate;

model Y = A | C1 | C2/link=id d=b;

output out = dsn_boot1_RD p=Ypred;

ods output ParameterEstimates=dsn_boot_parm_RD (keep = Replicate Parameter Estimate);

**run**;

ods listing;

**proc** **transpose** data=dsn_boot_parm_RD out=wideparm_RD (drop = _NAME_ bRD_Scale) prefix=brd_;

by replicate;

id parameter;

var estimate;

**run**;

**proc** **sort** data=wideparm_RD; by replicate; **run**;

**proc** **sort** data=dsn_boot1_RD; by replicate; **run**;

**data** gc_boot_RD;

merge dsn_boot1_RD wideparm_RD;

by replicate;

**proc** **sort** data=gc_boot_RD; by replicate; **run**;

/*

Step 2: Compute two predicted potential outcomes under treatment and no treatment for every individual

Step 3: Take the difference between the two potential outcomes under treatment and no treatment

*/

**Data** gc_boot1_RD;

set gc_boot_RD;

/*generate potential outcome*/

if A = **1** then Y1 = Ypred; /*Observed outcome, consistency*/

if A = **1** then Y0 = brd_Intercept + brd_A***0** + brd_C1*C1 +

brd_C2*C2 + brd_A_C2***0***C2 +

brd_A_C1***0***C1 + brd_C1_C2*C2*C1 +

brd_A_C1_C2***0***C2*C1; /*Counterfactual outcome, exchangeability*/

if A = **0** then Y0 = Ypred; /*Observed outcome, consistency*/

if A = **0** then Y1 = brd_Intercept + brd_A***1** + brd_C1*C1 +

brd_C2*C2 + brd_A_C2***1***C2 +

brd_A_C1***1***C1 + brd_C1_C2*C2*C1 +

brd_A_C1_C2***1***C2*C1; /*Counterfactual outcome, exchangeability*/

if A = **1** then ATT_RD = Y1 - Y0;

if A = **0** then ATU_RD = Y1 - Y0;

/*ATE_RD*/

ATE_RD = Y1 - Y0;

**run**;

/*

Step 3 (cont.): Summarize the contrast across the bootstrap samples

*/

**proc** **univariate** data=gc_boot1_RD noprint;

by replicate;

var ATT_RD ATU_RD ATE_RD;

output out=gc_boot2_RD

mean = ATT_RD ATU_RD ATE_RD ;

**run**;

**proc** **univariate** data=gc_boot2_RD noprint;

var ATT_RD ATU_RD ATE_RD;

output out=results_RD pctlpts=**2.5** **50** **97.5**

pctlpre = ATT_RD_p ATU_RD_p ATE_RD_p

mean = ATT_RD_m ATU_RD_m ATE_RD_m

std = ATT_RD_sd ATU_RD_sd ATE_RD_sd ;

**run**;

**Proc** **print** data=results_RD;

Title "ATT_RD";

var ATT_RD_m ATT_RD_sd ATT_RD_p2_5 ATT_RD_p97_5;

**Proc** **print** data=results_RD;

Title "ATU_RD";

var ATU_RD_m ATU_RD_sd ATU_RD_p2_5 ATU_RD_p97_5;

**Proc** **print** data=results_RD;

Title "ATE_RD";

var ATE_RD_m ATE_RD_sd ATE_RD_p2_5 ATE_RD_p97_5;

**run**;

### /*****ODDS RATIO*****/

/*

Step 1: Use bootstrap to resample the original data set 1000 times

*/

**proc** **surveyselect** data=dsn out=dsn_boot_OR

seed = **3321** method = urs

samprate = **1** outhits rep = **1000**;

**run**;

**proc** **sort** data=dsn_boot_OR; by replicate id; **run**;

/*

Step 1: Fit a flexible model for Y on A and covariates C for each bootstrapped sample

*/

ods listing close;

**proc** **genmod** data=dsn_boot_OR desc;by replicate;

model Y = A | C1 | C2/link=logit d=bin;

output out = dsn_boot1_OR p=Ypred;

ods output ParameterEstimates=dsn_boot_parm_OR (keep = Replicate Parameter Estimate);

**run**;

ods listing;

**proc** **transpose** data=dsn_boot_parm_OR out=wideparm_OR (drop = _NAME_ bOR_Scale) prefix=bor_;

by replicate;

id parameter;

var estimate;

**proc** **sort** data=wideparm_OR; by replicate; **run**;

**data** gc_boot_OR;

merge dsn_boot1_OR wideparm_OR;

by replicate;

**proc** **sort** data=gc_boot_OR; by replicate; **run**;

/*

Step 2: Compute two predicted potential outcomes under treatment and no treatment for every individual

Step 3: Calculate the odds ratio comparing potential outcomes under treatment versus no treatment

*/

**Data** gc_boot1_OR;

set gc_boot_OR;

/*generate potential outcomes*/

if A = **1** then Y1 = Ypred; /*Observed outcome, consistency*/

if A = **1** then Y0 = (**1**/(**1** + exp(-(bor_Intercept + bor_A***0** + bor_C1*C1 +

bor_C2*C2 + bor_A_C2***0***C2 +

bor_A_C1***0***C1 + bor_C1_C2*C2*C1 +

bor_A_C1_C2***0***C2*C1)))); /*Counterfactual outcome, exchangeability*/

if A = **0** then Y0 = Ypred; /*Observed outcome, consistency*/

if A = **0** then Y1 = (**1**/(**1** + exp(-(bor_Intercept + bor_A***1** + bor_C1*C1 +

bor_C2*C2 + bor_A_C2***1***C2 +

bor_A_C1***1***C1 + bor_C1_C2*C2*C1 +

bor_A_C1_C2***1***C2*C1)))); /*Counterfactual outcome, exchangeability*/

if A = **1** then ATT_OR = (Y1/(**1**-Y1))/(Y0/(**1**-Y0));

if A = **0** then ATU_OR = (Y1/(**1**-Y1))/(Y0/(**1**-Y0));

/*ATE_OR*/

ATE_OR = (Y1/(**1**-Y1))/(Y0/(**1**-Y0));

**run**;

/*

Step 3 (cont.): Summarize the contrast across the bootstrap samples

*/

**proc** **univariate** data=gc_boot1_OR noprint;

by replicate;

var ATT_OR ATU_OR ATE_OR;

output out=gc_boot2_OR

mean = ATT_OR ATU_OR ATE_OR ;

**run**;

**proc** **univariate** data=gc_boot2_OR noprint;

var ATT_OR ATU_OR ATE_OR;

output out=results_OR pctlpts=**2.5** **50** **97.5**

pctlpre = ATT_OR_p ATU_OR_p ATE_OR_p

mean = ATT_OR_m ATU_OR_m ATE_OR_m

std = ATT_OR_sd ATU_OR_sd ATE_OR_sd;

**run**;

**Proc** **print** data=results_OR;

Title "ATT_OR";

var ATT_OR_m ATT_OR_sd ATT_OR_p2_5 ATT_OR_p97_5;

**Proc** **print** data=results_OR;

Title "ATU_OR";

var ATU_OR_m ATU_OR_sd ATU_OR_p2_5 ATU_OR_p97_5;

**Proc** **print** data=results_OR;

Title "ATE_OR";

var ATE_OR_m ATE_OR_sd ATE_OR_p2_5 ATE_OR_p97_5;

**run**;

/******************* End of G-computation resampling *****************/
